# Chromosome-Scale Genome Assembly of Two Australian Nannochloropsis oceanica Isolates Exhibiting Superior Lipid Characteristics

**DOI:** 10.1128/MRA.01288-19

**Published:** 2019-11-27

**Authors:** Reuben B. Brown, Taylor J. Wass, Skye R. Thomas-Hall, Peer M. Schenk

**Affiliations:** a Algae Biotechnology Laboratory, School of Agriculture and Food Sciences, The University of Queensland, Brisbane, Queensland, Australia; University of California, Riverside

## Abstract

Nannochloropsis oceanica strains BR2 and KB1 are microalgal isolates from brackish water in the Brisbane River and a coastal rock pool at the Sunshine Coast in Australia which display superior productivity at high temperatures. We used long-read sequencing to sequence their genomes and to facilitate elucidation of loci associated with these traits.

## ANNOUNCEMENT

The model oleaginous eustigmatophyte microalga Nannochloropsis oceanica is used in industrial microalgal oil production, with lipid yields reported up to 70% dry weight (1). *Nannochloropsis* subsp. are primary producers of omega-3 long-chain polyunsaturated fatty acids, a valuable biocommodity, and are reputed for their compact genomes accompanied by a massive dose expansion of genes related to lipid biosynthesis (2).

Strain BR2 was isolated from the Brisbane River (3, 4), and strain KB1 was isolated from a tidal coastal rock pool at Kings Beach, Sunshine Coast, in Australia (26.8010°S, 153.1410°E) by incubating 1 liter of filtrate (5-μm mesh) in 50-ml tubes of f/2 medium on an orbital shaker at 80 rpm, 25°C, and 120 μmol photons m^−2^ s^−1^ continuous illumination until growth was visible. Then, serial dilutions were made to obtain unialgal cultures (5, 6). Cultures were grown under aseptic conditions to propagate sufficient cells for DNA isolation. Bacterial contamination was reduced, and DNA extracted as per reference 7. DNA was quality checked (Nanodrop 2000 spectrophotometer; Thermo Fisher Scientific) and quantified (Qubit; Thermo Fisher Scientific).

A total of 1 μg of genomic DNA (gDNA) was used for library preparation, which was constructed using the ligation sequencing kit (LSK-109; Oxford Nanopore Technologies [ONT]), using half-volumes of LSK-109 reagents. Recommended concentrations were used following library pooling. Barcodes were BC01 and BC02 for BR2 and KB1, respectively. Thirteen cycles of PCR were used, following ONT specifications with a 12-min extension step. The pooled and barcoded library was sequenced using an ONT MinION sequencer on an R9.4D flow cell and base called using Guppy v3.1.5 (ONT). Default software settings were used unless otherwise specified. A total of 7.4 Gb of base-called reads were demultiplexed and adaptors removed using qcat v1.1.0, yielding 4.3 Gb of data corresponding to BC01 (BR2) and 2.8 Gb corresponding to BC02 (KB1) (https://github.com/nanoporetech/qcat). Reads shorter than 1 kb or with an average Q-score below 7 were removed using NanoFilt, leaving 2.66 Gb (*N*_50_, 2,378 bp) and 1.20 Gb (*N*_50_, 2,761 bp) attributable to BC01 and BC02, respectively (https://github.com/wdecoster/nanofilt). The clean reads were assembled using pomoxis v0.2.4 and Canu v1.8 independently, and a consensus assembly was then generated using Flye v2.6 (https://github.com/nanoporetech/pomoxis) (8, 9). Following each step, consensus contigs were created using one round of Racon v1.4.7 and medaka v0.9.2 (10) (https://github.com/nanoporetech/medaka). The data were assembled into 1,384 contigs for BR2 and 1,856 contigs for KB1 that were scaffolded with RaGOO v1.1 against the *N. oceanica* LAMB2011 (GenBank accession number GCA_004519485) genome, giving 32 chromosomes for both strains, including complete, circular mitochondrial (BR2, 38,000 bp; KB1, 38,007 bp) and plastidial (BR2, 122,624 bp; KB1, 130,867 bp) sequences (11). The final genome sizes were 27.70 Mb (G+C content, 53.61%; 96× coverage; 1,111 gaps) and 27.05 Mb (G+C content, 53.54%; 44× coverage; 1,080 gaps) for BR2 and KB1, respectively. Average nucleotide identity was computed with OATu, with 99.4% (BR2) and 99.3% (KB1) similarity to LAMB2011 (12). Thus, the isolates were assigned to the species *Nannochloropsis oceanica*.

### Data availability.

Assemblies were deposited under the GenBank accession numbers GCA_009014725 (BR2) and GCA_009014695 (KB1). The BioProject accession number is PRJNA566059, and the SRA accession numbers are SRR10158724 (BR2) and SRR10158723 (KB1).
